# The Effect of Benzannulation on the Structures, Reactivity and Molecular Dynamics of Indenes, Pentalenes, Azulenes and Related Molecules

**DOI:** 10.3390/molecules27123882

**Published:** 2022-06-17

**Authors:** Michael J. McGlinchey

**Affiliations:** School of Chemistry, University College Dublin, Belfield, D04 V1W8 Dublin 4, Ireland; michael.mcglinchey@ucd.ie

**Keywords:** isoindenes, sigmatropic and haptotropic migrations, organometallic molecular brake, dibenzpentalenes, benzyne cycloadditions, benzazulenes, tetracenes, X-ray crystallography

## Abstract

The stabilising effect of benzannulation on isoindenes formed in the course of sigmatropic shifts of (C_5_H_5_)Fe(CO)_2_ or of organo-silyl groups, and on exocyclic allyl intermediates in the course of haptotropic shifts of organometallic fragments over polycyclic skeletons (fluorene, cyclopenta[*def*]phenanthrene, *syn* and *anti* dibenzpentalenes) is exemplified. This approach led to the development of the first organometallic molecular brake. Benzyne cycloadditions to anthracenes to form triptycenes also led to unexpected or multiple adducts that were characterised by X-ray crystallography. Synthetic routes to the previously elusive benz[*cd*]azulene system are presented. Finally, the complete mechanism of the stepwise assembly of dispiro- and diindenyltetracenes from fluorenylallenes is presented, whereby every intermediate has been unambiguously structurally characterised.

## 1. Introduction

Benzannulation, and its consequences for the newly formed molecule, have inspired synthetic and mechanistic investigations for decades. It can bring about perturbation of the molecular geometry, enhanced reactivity, the induction of chirality, and can also allow the observation of fascinating and novel molecular rearrangements. The major contributions of Erich Clar to the field of linear acenes, e.g., tetracene or pentacene, and their angular counterparts, such as phenanthrene or triphenylene, with their different reactivity patterns, especially in cycloadditions, are well recognised; thus, benzynes react with anthracenes to furnish triptycenes with their varied and resplendent properties. Moreover, the concept of the aromatic 6π sextet has found widespread applicability in acenes and in polycyclic aromatics [1].

## 2. The Indenyl Effect

**Scheme 1:**
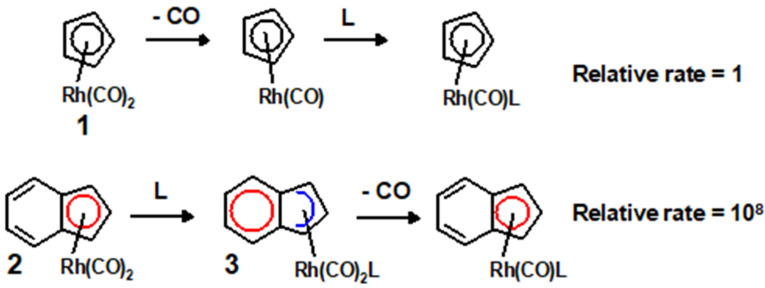
The indenyl effect exemplified in rhodium complexes.

A dramatic example of the effect of benzannulation on reactivity is provided by the so-called “indenyl effect” as reported by the distinguished inorganic chemist Fred Basolo in 1984 [2]. Substitution of a carbonyl ligand by a phosphine in (η^5^-cyclopentadienyl)dicarbonylrhodium(I), **1**, proceeds in a stepwise manner, via dissociative loss of a CO with subsequent addition of the phosphine to the 16-electron centre. In contrast, in the corresponding indenyl complex, **2**, the reaction exhibits second-order kinetics, first-order in both the rhodium complex and the phosphine. In this bimolecular process, attack of the incoming phosphine is facilitated by slippage of the rhodium towards the η^3^ structure, **3**, thus maintaining an 18-electron configuration at the metal, but also inducing full aromatic character in the six-membered ring (Scheme 1). The net result is a dramatic reduction in the activation energy for the process, and an enormous rate enhancement of 10^8^ for the indenyl system relative to its cyclopentadienyl counterpart. Since that time, the proposed indenyl effect has been fully vindicated, and the slip-fold η^3^ geometry of the indenyl ligand has been unequivocally established by X-ray crystallography [3].

## 3. Enhancement of Aromatic Character by Benzannulation

### 3.1. Stabilisation of Isoindene Intermediates in 1,5 Sigmatropic Migrations

**Scheme 2:**
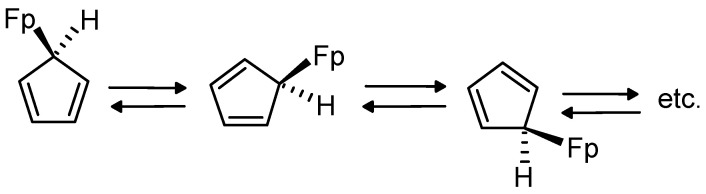
Metallotropic shifts around a cyclopentadiene ring, where Fp = (C_5_H_5_)Fe(CO)_2_.

While the whole area of the syntheses and structures of polybenzannulated homologues of cyclopentadiene, and their organometallic derivatives, has been comprehensively reviewed [4], we focus here on their molecular dynamics. The inherent chirality engendered by the benzannulation of monosubstituted cyclopentadienes has allowed the elucidation of a number of molecular rearrangements. Indeed, this phenomenon was a factor in the very first reported example of fluxional behaviour in organometallic chemistry. When bis(cyclopentadienyl)dicarbonyliron, (C_5_H_5_)_2_Fe(CO)_2_, was prepared by Piper and Wilkinson in 1956, its ^1^H NMR spectrum exhibited only two equally intense singlet peaks [5]. A subsequent variable-temperature NMR study by a group at MIT revealed that at low temperature, one of these signals decoalesced to exhibit a 1:2:2 pattern that was interpreted in terms of a structure in which one of the cyclopentadienyl rings was π-bonded, whereas in the other ring, only a single carbon was σ-linked to the iron atom, a proposal that was confirmed by X-ray crystallography [6]. As shown in Scheme 2, it was eventually established that the (η^5^-C_5_H_5_)FeCO)_2_ moiety (Fp) was undergoing a series of [1,2] migrations (subsequently reformulated in Woodward–Hoffmann terms as [1,5]-suprafacial sigmatropic shifts) with a barrier of 45 kJ mol^−1^.

**Scheme 3:**
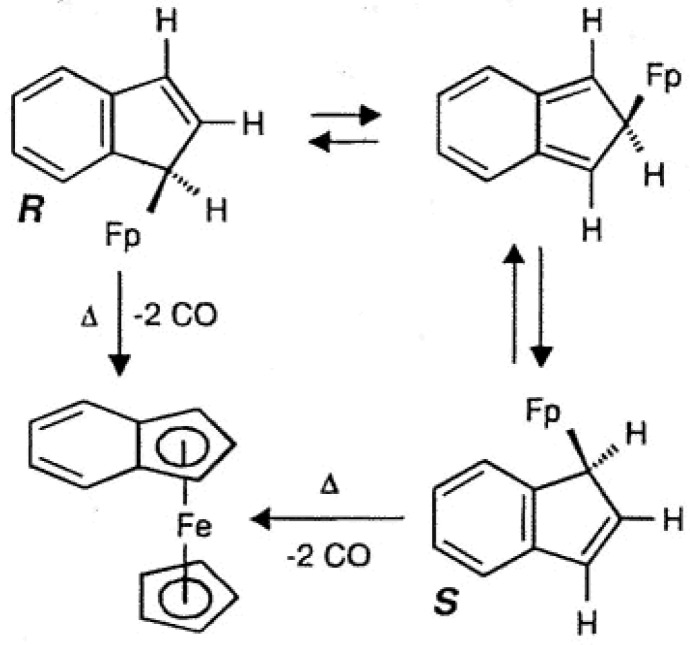
Verification of the intermediacy of an isoindene was thwarted by decarbonylation.

However, when one ligand was replaced by an indenyl substituent, as in Scheme 3, the corresponding fluxional process could not be observed, perhaps indicating that if such a [1,5] shift were to occur, this would involve the intermediacy of an isoindene with partial disruption of aromaticity, and presumably a substantially increased barrier to migration. Moreover, attempts to resolve this question by raising the temperature to 70 °C merely brought about decomposition with loss of the carbonyl ligands and formation of benzoferrocene [7].

**Scheme 4:**
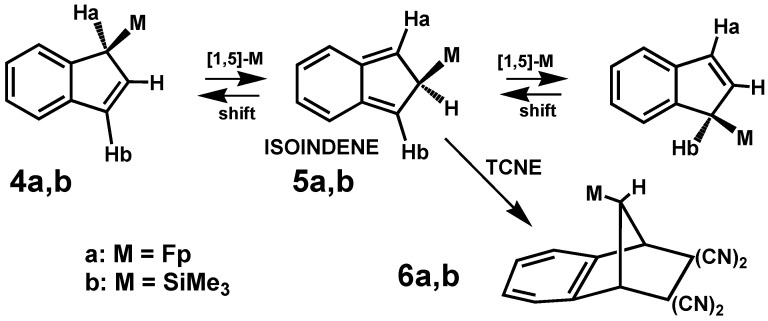
Metallotropic [1,5] shifts of organo-iron and organo-silicon moieties, and Diels–Alder trapping of the isoindene intermediates with tetracyanoethylene.

The situation was eventually resolved 30 years later by taking advantage of the development of the 2D EXSY NMR technique that facilitates the observation of chemical exchange processes without the need to raise the temperature such that line broadening is evident, and which revealed a markedly increased barrier of ~85 kJ mol^−1^. Conclusive evidence for the intermediacy of the isoindene, **5a**, was provided by its reaction with tetracyanoethylene (TCNE) at room temperature to form the Diels–Alder adduct, **6a**, that was characterised by X-ray crystallography (Scheme 4) [8].

**Figure 1:**
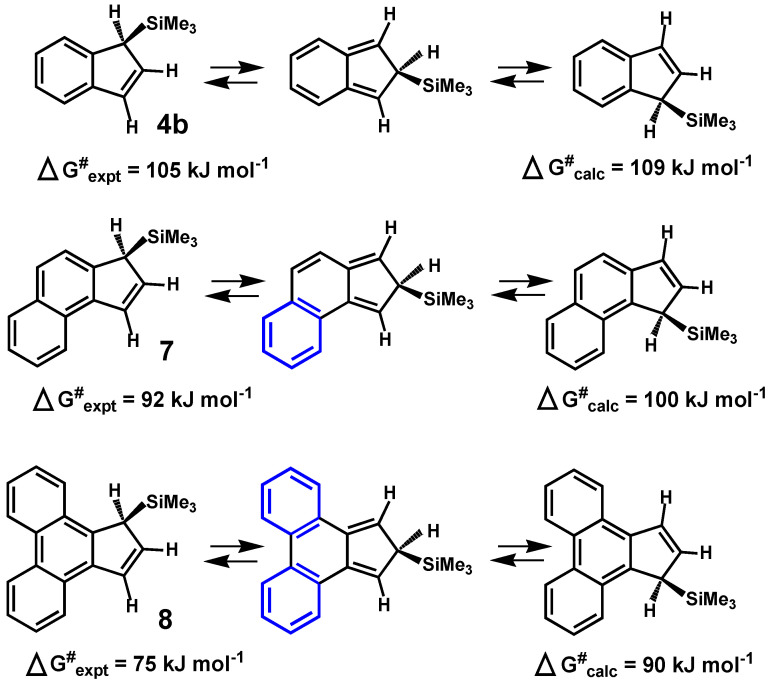
Calculated and experimental barriers to silatropic shifts in a series of benzindenes.

Closely analogous behaviour is also found in the corresponding trimethylsilylindene, **4b**, whereby successive [1,5] suprafacial sigmatropic shifts interconvert enantiomers via the isoindene intermediate, **5b**, that was again trapped as its TCNE Diels–Alder adduct, **6b** [9]. Interestingly, it was also demonstrated that the incorporation of additional benzo rings, as in Figure 1, to extend the aromatic framework from 6π to 10π to a 14π electron system markedly lowers the barrier to migration via an isoindene intermediate, apparently by enhancement of the aromatic character of the intermediate isoindenes. Calculations at the unrestricted Hartree–Fock level yielded a barrier of 109 kJ mol^−1^ for trimethylsilylindene, **4b**, 100 kJ mol^−1^ for the angular trimethylsilylbenzindene, **7**, and 90 kJ mol^−1^ for the dibenzindene (trimethylsilylcyclopenta[*l*]phenanthrene), **8**. Gratifyingly, the experimentally determined migration barriers correlate well with theoretical predictions [10,11].

**Figure 2:**
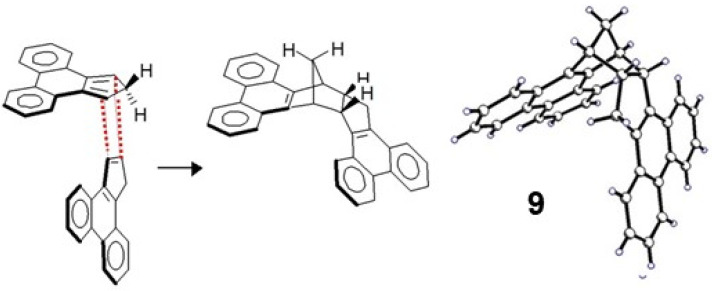
Molecular structure of the Diels–Alder dimer, **9**, formed by reaction of cyclopenta[*l*]phenanthrene with its own isoindene.

This latter result provides an ideal rationale for the observation that, when cyclopenta[*l*]phenanthrene was heated at reflux in dibutyl ether, the product (85% yield) was the dimer, **9**, whose X-ray crystal structure is shown in Figure 2 [12]. Evidently, the isoindene generated by thermolysis of dibenz[*e*,*g*]indene is sufficiently long-lived that it undergoes Diels–Alder cycloaddition with its own precursor.

**Scheme 5:**
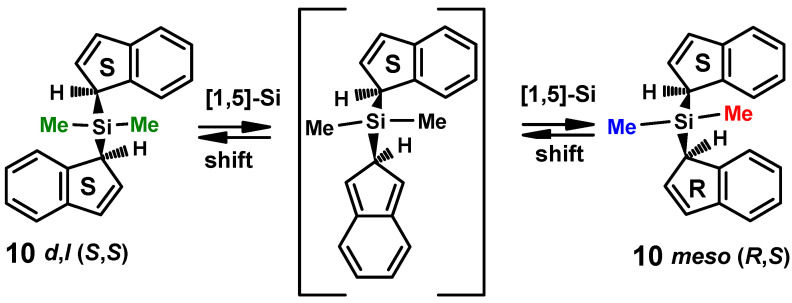
Interconversion of *d,l* and *meso* diastereomers of bis(indenyl)dimethylsilane, **10**.

This project has been extended to encompass molecules bearing multiple η^1^-indenyl substituents, as in bis(indenyl)dimethylsilane, **10**, tris(indenyl)methylsilane, **11**, and even tetrakis(indenyl)- silane, -germane and -stannane [13,14]. We see from Scheme 5 that in bis(indenyl)dimethylsilane, consecutive [1,5] shifts, whereby a silicon migrates from C(1) via C(2) to C(3), interconvert diastereomers. One should note that the methyl groups in the *C*_2_ isomers (*S*,*S*) and (*R*,*R*) are symmetry equivalent, whereas in the *meso* compound (*R*,*S*), they are rendered non-equivalent since they lie in the molecular mirror plane and appear as separate NMR singlets. Monitoring exchange between these methyl environments allowed evaluation of the migration barrier as ~96 kJ mol^−1^ and, gratifyingly, the system gave rise to a double Diels–Alder adduct with TCNE [15].

**Scheme 6:**
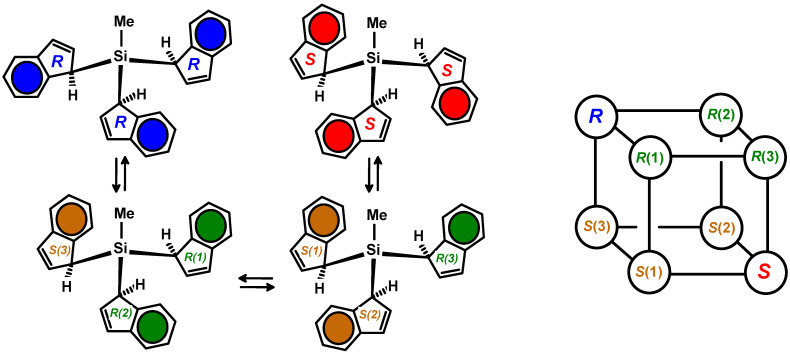
(**Left**) Interconversions of the eight different indenyl ring environments of tris(indenyl)methylsilane. The configurational inversion of a single indenyl ring requires two [1,5]-suprafacial sigmatropic shifts [9]. (**Right**) The cube representing exchange between the eight different ring environments in **11**.

We focus here briefly on tris(indenyl)methylsilane, **11**, which gives rise to *RRR*, *RRS*, *RSS* and *SSS* isomers in a 1:3:3:1 ratio, where the *R* and *S* labels refer to the absolute configuration of C(1) in each indenyl ring, as shown in Scheme 6.

**Scheme 7:**
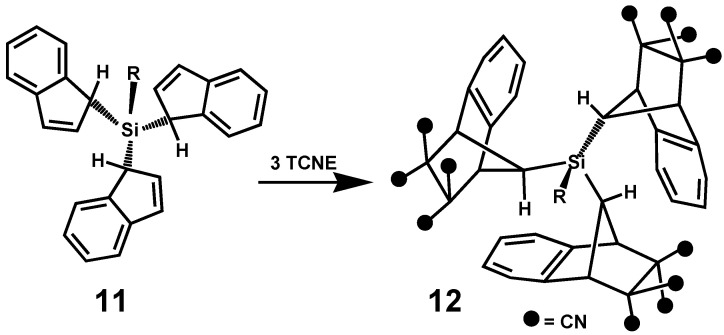
Multiple Diels–Alder reactions with TCNE to form the triple adduct **12**.

The full gamut of two-dimensional ^1^H, ^13^C and ^29^Si NMR data revealed the unequivocal assignment of the proton, carbon-13 and silicon-29 nuclei in all of the different indenyl ring environments and ultimately allowed the elucidation of their molecular dynamics [9]. As depicted in Scheme 6, the exchange pathways between indenyl sites in **11** can be mapped onto a cube (for the eight different indenyl ring environments), and a hypercube (for the exchange of ^1^H in sp^2^ and sp^3^ environments) and again involve successive [1,5]-suprafacial sigmatropic shifts via isoindene intermediates; gratifyingly, several triple Diels–Alder TCNE adducts, **12**, have been isolated and fully characterised by X-ray crystallography (Scheme 7) [16,17].

We note in passing that the chiral character of the indenyl unit has also been exploited for catalytic purposes. Typically, the *C*_2_-symmetric dichlororo-dibenzozirconocene, **13**, functions as a Ziegler–Natta-type polymerisation catalyst for the stereospecific formation of isotactic polypropylene [18].


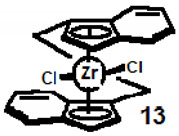


### 3.2. Lowering the Barriers to Migrations in Haptotropic Shifts

#### 3.2.1. Metal Complexes of Cyclopenta[*def*]phenanthrene

Polycyclic systems bearing π-bonded organometallic moieties frequently exhibit haptotropic shifts whereupon the metal migrates between rings either thermally, or under basic or acidic conditions [19]. As elucidated on the basis of molecular orbital calculations, notably by Albright and Hoffmann [20], the most direct route across the common bond between the rings generally has a symmetry-imposed high activation energy, and so the pathway adopted is rather circuitous. Moreover, an electron-counting rule was advanced such that when the total number of electrons supplied by the ligand and the metal equals 4*q* + 2, where *q* = 2, 3, …, then haptotropic shifts passing directly below the common bond are forbidden. However, when this sum equals *q*, the process is partially allowed, and the trajectory of the rearrangement deviates less strongly from the least-motion pathway.

**Scheme 8:**
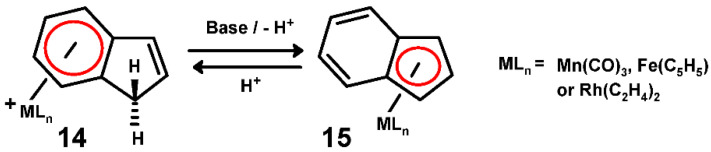
Haptotropic shifts in the indene system; ML_n_ = Mn(CO)_3_, Fe(C_5_H_5_) or Rh(C_2_H_4_)_2_.

Typically, as illustrated in Scheme 8, upon deprotonation of a cationic complex, such as [(η^6^-indene)manganesetricarbonyl]^+^, **14**, the organometallic tripod undergoes an η^6^ → η^5^ (hexahapto to pentahapto) migration to form the neutral species **15** [21]. However, we focus here specifically on the migration pathway that has been shown to proceed via an exocyclic η^3^-allylic transition state rather than by the least-motion shortest path between the ring centres. In many cases, upon protonation, this process is reversible, i.e., pentahapto to hexahapto; interestingly, at least in the rhodium case, the initial protonation appears to occur at the metal centre since use of D^+^ rather than H^+^, brings about deuteration of the ethylene ligands [22].

**Scheme 9:**
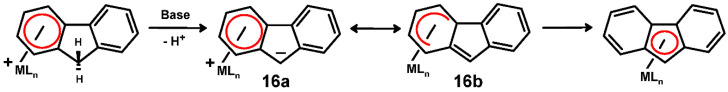
A hexahapto to pentahapto shift in the fluorene system.

The phenomenon of haptotropic migration has been observed in numerous other polycyclic frameworks, in particular the next benzannulated homologue, fluorene. In this case, deprotonation yields initially a zwitterionic intermediate, **16a**, which is perhaps better described as having an η^5^-bonded metal and an exocyclic double bond, as in **16b**. As depicted in Scheme 9, subsequent migration leads eventually to the final η^5^-fluorenyl product [23].

**Figure 3:**
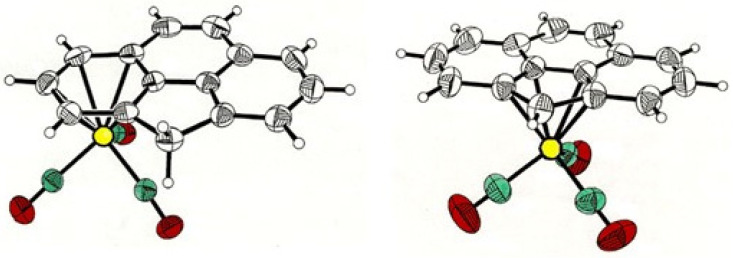
X-ray crystal structures of (**left**) (η^6^-cppH)Cr(CO)_3_ and (**right**) (η^5^-cpp)Mn(CO)_3_.

Of direct relevance to our current focus on the effects of benzannulation, we note that the 4*H*-cyclopenta[*def*]phenanthrene (cppH) skeleton can be considered to contain the carbon skeletons of indene, acenaphthylene, phenanthrene and fluorene, and that the syntheses and structures of a number of its organometallic complexes have been reported [24]. Typically, the X-ray crystal structures of the (η^6^-cppH)Cr(CO)_3_ and (η^5^-cpp)Mn(CO)_3_ complexes are shown as Figure 3.

**Scheme 10:**
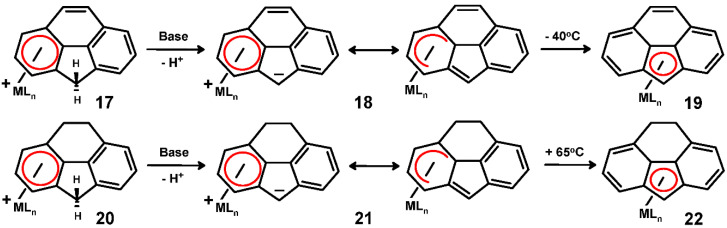
Hexahapto to pentahapto shifts in the 4*H*-cyclopenta[*def*]phenanthrene (cppH) system.

**Figure 4:**
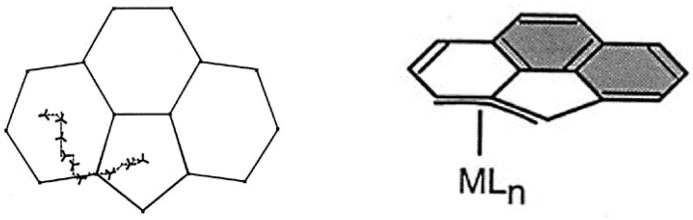
The calculated trajectory of an η^6^ to η^5^ haptotropic shift taken by an Mn(CO)_3_ tripod via an η^3^ transition state apparently stabilised by development of aromatic character in the (shaded) naphthalene fragment.

Deprotonation of the [(η^6^-cppH)Mn(CO)_3_]^+^ cation, **17**, yields initially the (η^5^-cpp)Mn(CO)_3_ species, **18**, but in this case it readily undergoes a haptotropic shift, even at −40 °C, to form **19** (Scheme 10). In marked contrast, in the 8,9-dihydro-cppH analogue, **20**, the initially formed (η^5^-dihydrocpp)Mn(CO)_3_ species, **21**, is isolable and resists migration to yield **22** until being held at 60 °C in hexane for an hour, thus paralleling the behaviour of its indene analogue. One may thus conclude that the lowered barrier to haptotropic migration in **17** is attributable to the presence of the 10π naphthalene moiety in the transition state, perhaps analogous to the aromatic stabilisation invoked in Basolo’s indenyl effect. The calculated trajectory taken by a metal tricarbonyl moiety in the course of a hexahapto to pentahapto rearrangement appears as Figure 4 [25].

**Figure 5:**
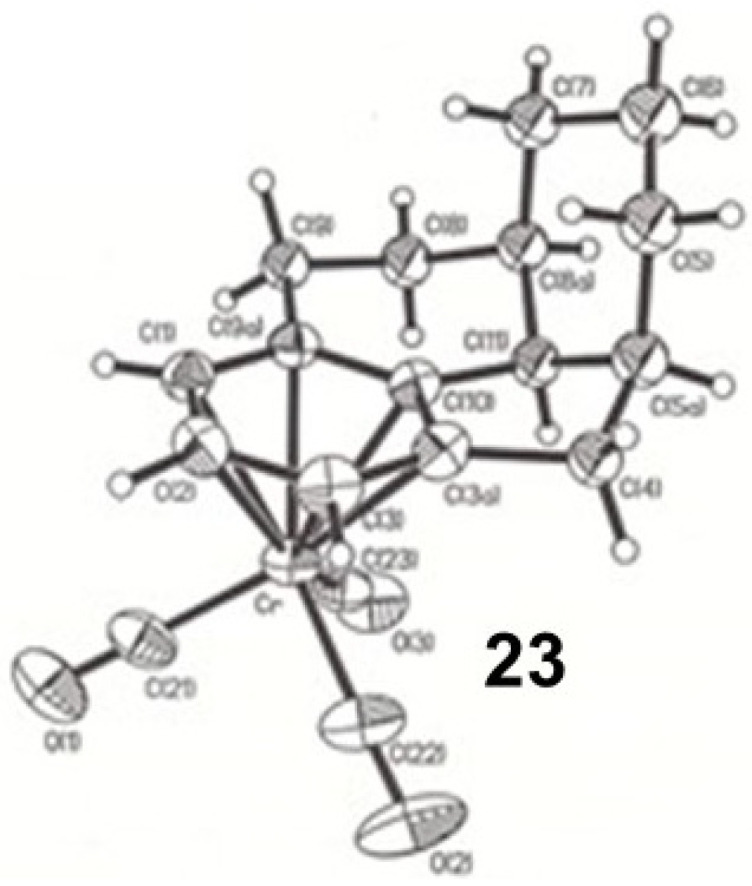
Molecular structure of (η^6^-octahydro-cpp)Cr(CO)_3_, **23**.

We note in passing that, during the preparation of (η^6^-dihydro-cpp)Cr(CO)_3_, further reduction of the ligand led to traces of (η^6^-octahydro-cpp)Cr(CO)_3_, **23**, in which only the complexed aromatic ring is unaffected. The resulting X-ray crystal structure appears as Figure 5, and reveals that the other external six-membered ring exhibits a chair conformation, while the internal ring is a half-chair, and the five-membered ring, as usual, adopts an envelope conformation [26].

**Scheme 11:**
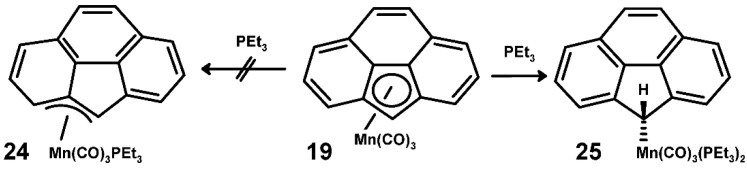
Attempted preparation of (η^3^-cpp)Mn(CO)_2_(PEt_3_) led to (η^1^-cpp)Mn(CO)_3_(PEt_3_)_2_.

First attempts to trap the proposed exocyclic allyl-type (η^3^-cpp)Mn(CO)_3_ species led to unexpected, but interesting results (Scheme 11). Careful addition of triethylphosphine to (η^5^-cpp)Mn(CO)_3_, **19**, with the goal of isolating (η^3^-cpp)Mn(CO)_3_PEt_3_, **24**, instead induced a different change in hapticity to form trans-(η^1^-cpp)Mn(CO)_3_(PEt_3_)_2_, **25**, the first σ-bonded cpp-ML_n_ system to have been structurally characterised [25].

**Scheme 12:**
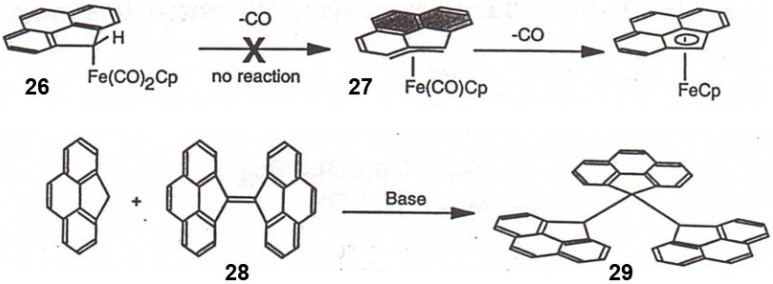
Attempted preparation of (η^3^-cpp)Fe(CO)(η^5^-C_5_H_5_) led to the cpp dimer, **28**, and trimer, **29**.

**Figure 6:**
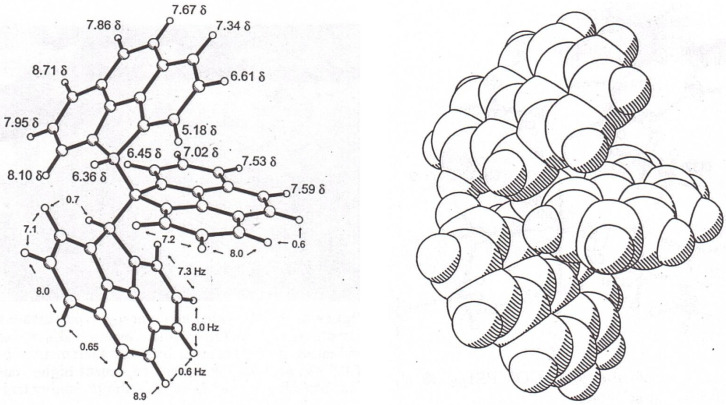
^1^H NMR spectroscopic assignments, and a space-fill view of **29**.

Another approach involved the initial preparation of (η^1^-cpp)Fe(CO)_2_(η^5^-C_5_H_5_), **26**, in the hope that Me_3_NO-induced loss of a single carbonyl ligand would result in formation of allylic (η^3^-cpp)Fe(CO)(η^5^-C_5_H_5_), **27**. Experimentally, under the basic conditions of the synthesis of **26**, fragmentation of the cpp-Fe linkage yielded the fulvalene, **28**, and a cpp trimer, **29** (Scheme 12). The 500 MHz ^1^H NMR spectrum of **29** revealed the non-equivalence of every proton within each of the two external cpp fragments, implying a sterically crowded structure of *C*_2_ symmetry, as depicted in the space-filling view shown in Figure 6 [25].

Success was finally achieved by researchers in a Portuguese/German collaboration who isolated and structurally characterised (η^3^-cpp)Mo(CO)_2_(η^5^-C_9_H_7_), **30**. This structure was also found to be in accord with predictions at the DFT level of calculations [27].


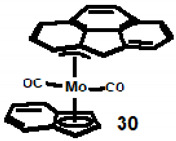


#### 3.2.2. Metal Complexes of *syn* and *anti* Dibenzopentalene

**Scheme 13:**
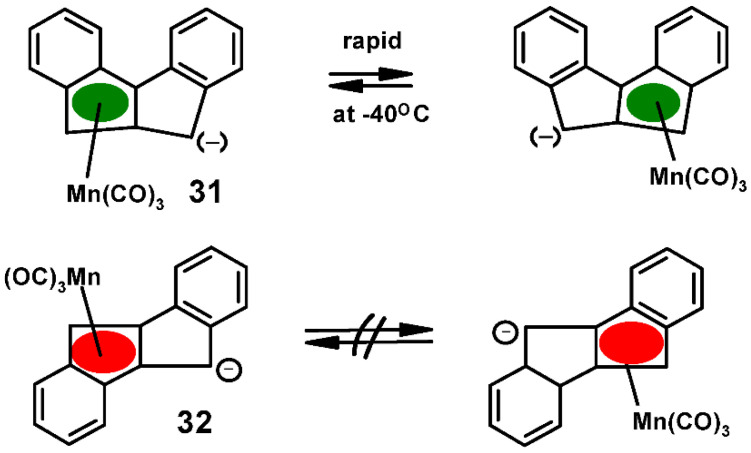
Very different migration behaviour in the *syn* and *anti* dibenzopentalenes, **31** and **32**.

In elegant work from Ustynuk’s group in Moscow, the rearrangement behaviour of metal complexes of 9,10-dihydroindeno[1,2-*a*]indene and 5,10-dihydroindeno[2,1-*a*]indene, more trivially known as *syn* and *anti* dibenzopentalenes, **31** and **32**, respectively, has been shown to be strikingly different (Scheme 13). In the *syn* isomer, η^5^ → η^5^ facile migration of the manganese tricarbonyl unit has been observed to occur with a barrier of ~65 kJ mol^−1^ [28]. In marked contrast, the corresponding *anti* isomer, **32**, resists migration, and we sought an explanation for this result. Clearly, since these molecules were isomeric, the *q* or 4*q* + 2 electron count discussed above should not be the controlling feature.

**Figure 7:**
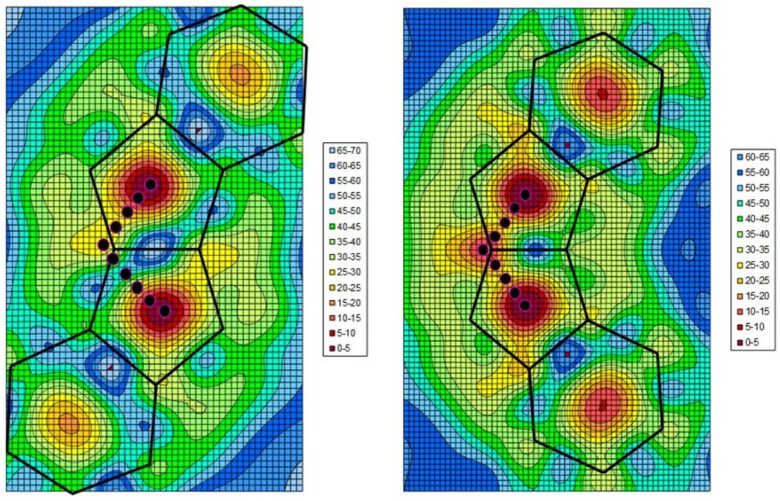
(**Left**) Potential energy hypersurface for the migration of an [Fe(C_5_H_5_)]^+^ fragment over the dianion of the *anti*-dibenzpentalene framework, **34**. Contour lines are incremented in units of 5 kcal mol^−1^, and the favoured reaction path is superimposed as a dotted line. (**Right**) As above, for the *syn*-dibenzpentalene system, **33**.

Molecular orbital calculations, initially of the Extended Hückel type, subsequently at the DFT level were carried out on the *syn* and *anti* iron complexes, **33** and **34**, respectively, and the resulting potential energy surfaces (PES) are shown in Figure 7 [29]. (The choice of an Fe(C_5_H_5_) moiety as the migrating group was to avoid complications arising from the changing orientation of the tripod during the transition between the rings.)


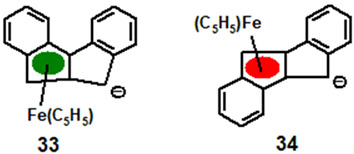


For the *anti* isomer, **34**, (Figure 7, left) the most obvious result is the greatly disfavoured ~72 kcal mol^−1^ (~300 kJ mol^−1^) least-motion pathway directly across the common bond between the five-membered rings. Moreover, the alternative route (dotted line on the PES) that bypasses the centre of this bond still requires that the migrating group surmount a barrier of ~45 kcal mol^−1^ (~188 kJ mol^−1^) and, as noted above, is not observed experimentally. In contrast, the PES for the *syn* isomer, **33**, (Figure 7, right) reveals a relatively low energy pathway whereby the migrating group passes almost directly below a ring junction carbon to yield an intermediate structure only 42 kJ mol^−1^ less stable than the *η^5^* minimum; the highest point on this route is only ~105 kJ above the ground state, considerably lower than that required for its *anti* counterpart, **34**.

**Scheme 14:**
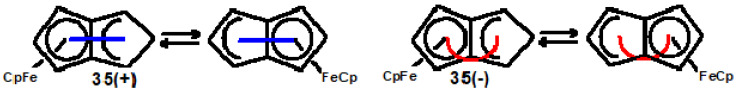
Migration pathway of a (C_5_H_5_)Fe unit across pentalene in its cationic and anionic forms.

In a later DFT study by Saillard, inter-ring migration of a (η^5^-C_5_H_5_)Fe moiety across the parent pentalene skeleton in both its cationic and anionic forms was investigated [30]. It was found that in the cation [(C_8_H_8_)Fe(C_5_H_5_)]^+^, **35(+)**, the process follows a least-motion (blue) pathway across the centre of the common bond between the rings, with a barrier of 184 kJ mol^−1^. In contrast, in the corresponding anion, **35(-)**, the preferred route (red) passes via an η^3^ intermediate (Scheme 14), analogous to the behaviour of its dibenzo counterpart. However, in the pentalene system, the computed overall migration barrier was found to be 164 kJ mol^−1^, markedly higher than the experimental value of ~65 kJ mol^−1^ observed by Ustynuk for the manganese complex, **31**. Evidently, the two external benzannulations have reduced the barrier for the η^5^ → η^5^ haptotropic shift rather substantially.

## 4. [4 + 2] Cycloadditions to Anthracenes and to 2-Phenylindene

### 4.1. Benzyne Addition to 3-Indenylanthracene and 2-Indenyl-anthracene

**Scheme 15:**
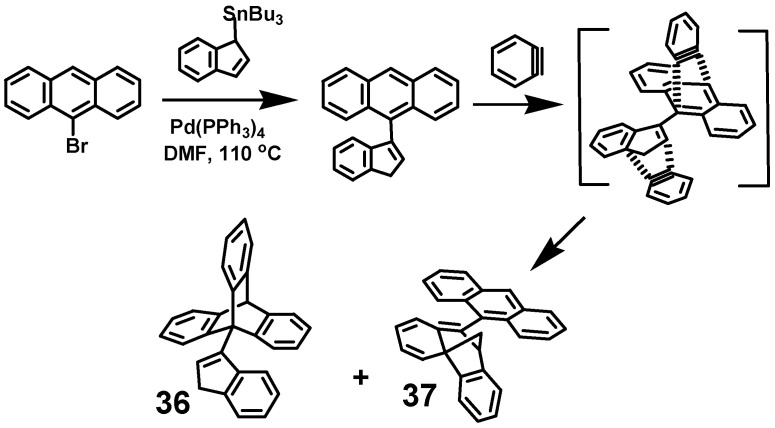
Palladium-catalysed route to 3-(9-indenyl)anthracene and its benzyne adducts **36** and **37**.

**Figure 8:**
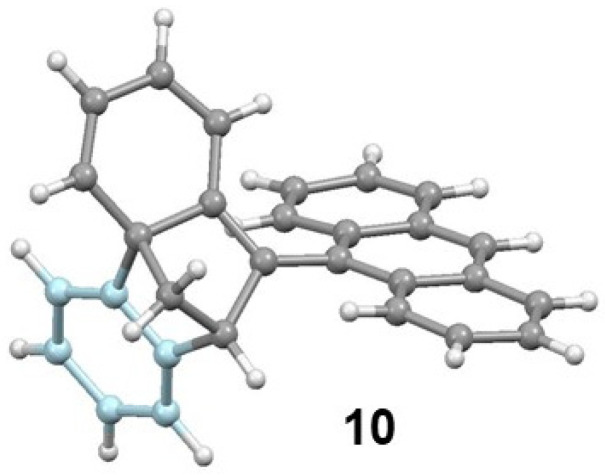
Molecular structure of benzyne adduct **37**.

Diels–Alder cycloadditions of benzynes to anthracenes are the standard route to triptycenes and, with the goal of preparing 9-(3-indenyl)triptycene, **36**, such a reaction was carried out. However, while the major product was the anticipated one, a second, unexpected isomer was also formed (Scheme 15). Benzyne added not only across C(9) and C(10) of the anthracene, but also to the indenyl substituent, thus forming the [4 + 2] cycloadduct, **37**, whose structure (Figure 8) was validated by X-ray crystallography [31].

**Scheme 16:**
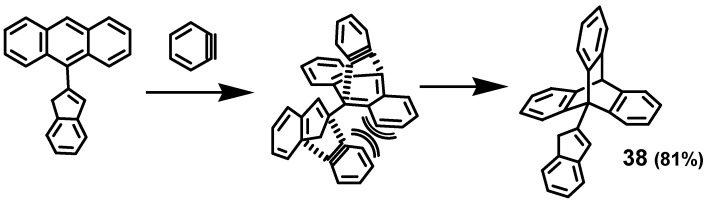
Reaction of benzyne and 9-2(indenyl)anthracene yields only a single adduct, **38**.

In contrast, benzyne and 2-indenylanthracene yield only 9-(2-indenyl)triptycene, **38**, in excellent yield. As depicted in Scheme 15 and Scheme 16, in 3-indenylanthracene, access of the incoming benzyne to both the anthracene and indenyl substituent is available, whereas in its 2-indenyl counterpart, the latter process would be blocked by the proximity of the planar anthracenyl unit, thus leading to a single product in enhanced yield.

### 4.2. Benzyne Addition to 2-Phenylindene

**Scheme 17:**
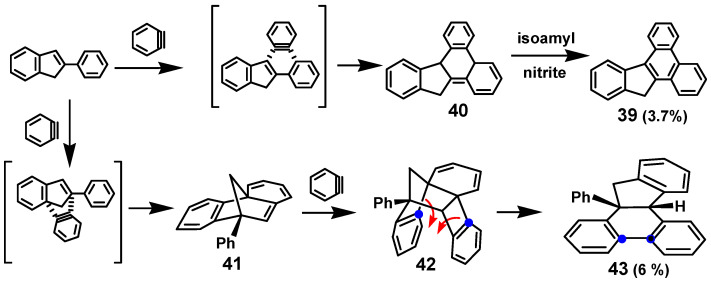
Cycloaddition reactions of benzyne to 2-phenylindene.

**Figure 9:**
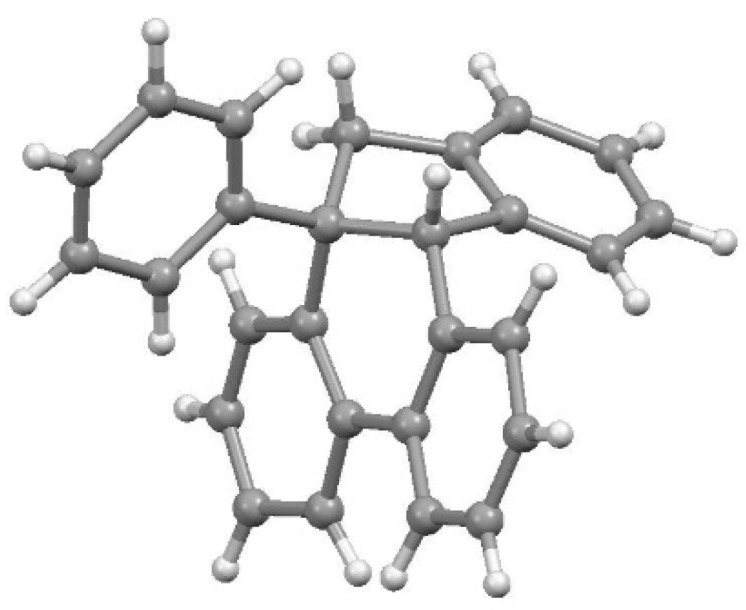
Molecular structure of **43**, the double benzyne adduct to 2-phenylindene.

In light of the unexpected observation of benzyne addition to the indenyl substituent of 3-indenylanthracene, its reaction with 2-phenylindene was investigated and yielded two products, but only in poor yields. However, their identities were established spectroscopically and by X-ray crystallography (Scheme 17 and Figure 9). The first product was characterised as the known indeno-anthracene, **39**, presumably resulting from the addition of a single benzyne to form the dihydroindenophenanthrene, **40**, that was further oxidised in the presence of excess isoamyl nitrite.

The formula of the second product corresponded to the addition of two benzyne units to phenylindene; one can envisage the first step as the [4 + 2] cycloaddition to the indenyl unit to form **41**, analogous to the reaction of benzyne with 9-(3-indenyl)anthracene to yield **37**. In the second step, [2 + 2] addition yields a cyclobutene, **42**, that adopts the *syn*, rather than the *anti*, configuration because of the presence of the adjacent phenyl substituent. Subsequent thermolysis can open up the four-membered ring and bring about rearrangement to the observed product **43**. The thermodynamic driving forces for such a process would be the relief of steric strain in the cyclobutene ring and the recovery of aromatic character in the original six-membered ring of the indene [31].

### 4.3. Benzyne Additions to Ferrocenylanthracenes

**Scheme 18:**
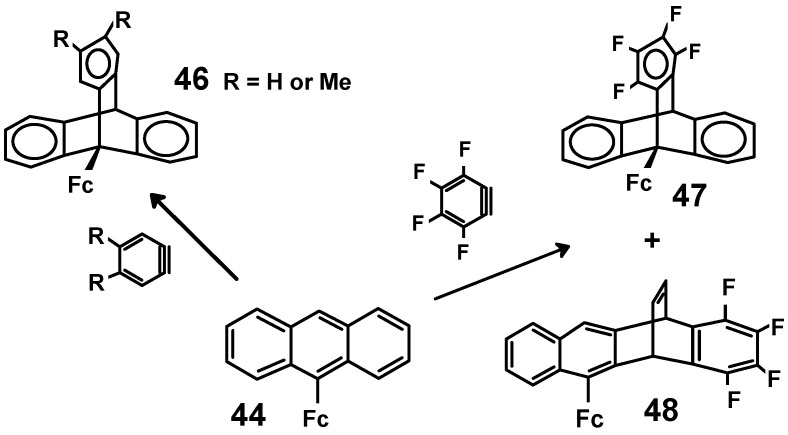
Reactions of benzynes with 9-ferrocenylanthracene.

**Scheme 19:**
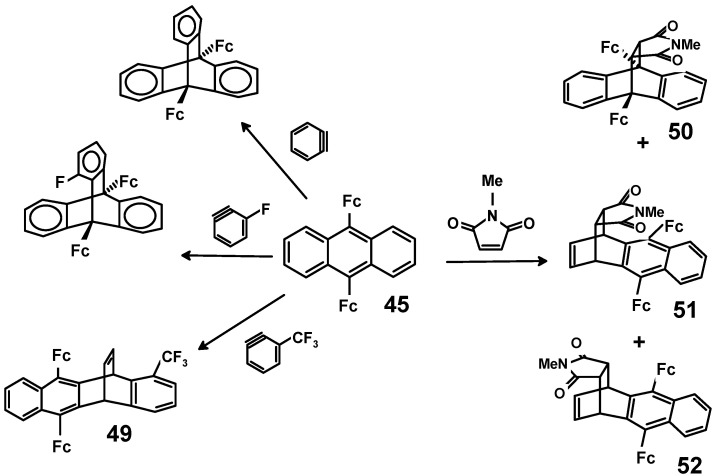
Reactions of benzynes or *N*-methylmaleimide with 9,10-diferrocenylanthracene.

As is well-established, benzynes add to anthracenes to form triptycenes. However, when bulky substituents are present, steric factors can come into play. Thus, 9-ferrocenylanthracene, **44**, and 9,10-diferrocenylanthracene, **45**, have been treated with a range of methyl or fluorobenzynes to give a multitude of products. Benzyne itself, and also 3-fluorobenzyne and 4,5-dimethylbenzyne, add to **44** to form the corresponding triptycenes, **46**, but tetrafluorobenzyne yields not only the conventional 9,10-adduct, **47**, but also undergoes Diels–Alder cycloaddition to a terminal ring, thereby producing 1,2,3,4-tetrafluoro-9-ferrocenyl-5,12-etheno-5,12-dihydrotetracene, **48** (Scheme 18). By way of contrast, while benzyne and 3-fluorobenzyne react with 9,10-diferrocenylanthracene to yield triptycenes, 3-trifluoromethylbenzyne reacts only to form the etheno-bridged tetracene, **49**. *N*-methylmaleimide gives three products, the 9,10-barrelene, **50**, and also both *endo* and *exo* 1,4-adducts, **51** and **52** (Scheme 19) [32].

### 4.4. The First Organometallic Molecular Brake

**Scheme 20:**
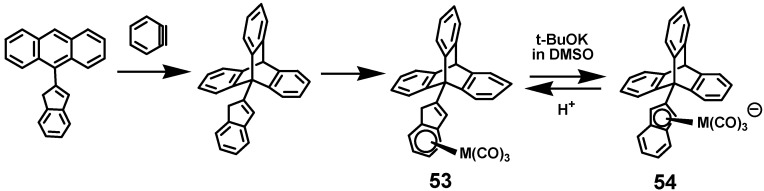
Deprotonation of **53** brings about an η^6^ **→** η^5^ haptotropic shift, forming **54** in which paddlewheel rotation is dramatically slowed on the NMR time-scale.

A reversible haptotropic shift is the key feature of an organometallic molecular brake whereby η^6^ → η^5^ migration of a metal carbonyl fragment across an indenyl framework brings about severely hindered rotation of a triptycene paddlewheel. As noted above, Diels–Alder cycloaddition of benzyne to 9-(2-indenyl)anthracene furnishes 9-(2-indenyl)triptycene, and subsequent incorporation of a metal tricarbonyl unit (ML_n_ = Cr(CO)_3_, or [M(CO)_3_]^+^, where M is Mn or Re) yields the hexahapto-bonded complexes **53**. In those cases, the triptycyl paddlewheel is free to rotate. However, deprotonation to form the corresponding pentahapto species, **54**, positions the organometallic fragment so as to block rotation of the triptycene paddlewheel (Scheme 20) [33]; X-ray data were obtained for several of these η^6^ and η^5^ complexes. This project, including many related Diels–Alder reactions, has been fully discussed elsewhere in this Journal [34].

## 5. Benzannulation as a Route to Non-Planar Polycyclic Hydrocarbons

### 5.1. Syntheses of Fragments of C_60_

**Figure 10:**
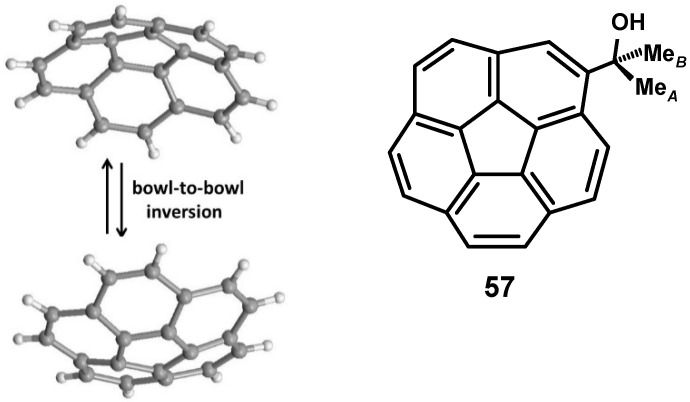
Inversion of corannulene, **55**, can be monitored via the diastereotopic methyls in **57**.

The evolving chemistry of fullerenes has prompted a number of approaches towards a logical, stepwise synthesis of C_60_. Major fragments of this molecule include corannulene, **55**, and sumanene, **56**. The former possesses a central pentagon surrounded by six-membered rings, whereas in the latter case a benzene moiety lies within a ring of alternating five- and six-membered rings. Most importantly, these molecules are non-planar and adopt bowl-shaped structures that bring about a change in symmetry from *D*_5h_ or *D*_3h_ to *C*_5v_ or *C*_3v_, respectively. However, umbrella-type inversion of **55** has been monitored by the incorporation of diastereotopic methyl groups as in corannulenyldimethylcarbinol, **57**, whereby their coalescence in the ^1^H or ^13^C variable-temperature NMR regimes functions as a probe of the activation energy (~43 kJ mol^−1^) for the racemisation of the resulting enantiomers (Figure 10) [35].

**Scheme 21:**
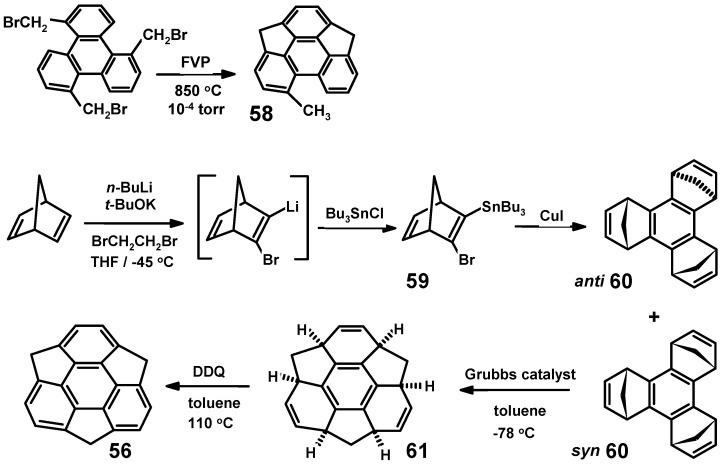
An early attempt, and the final and successful route to sumanene.

Initial attempts to prepare bowl-shaped sumanene from planar 1,5,9-tris(bromomethyl)triphenylene by flash vacuum pyrolysis (FVP) only yielded the doubly bridged product **58** [36]. Success was finally achieved by copper-mediated trimerization of 1-bromo-2-tributylstannylnorbornadiene, **59**, to give a 3:1 mixture of *anti* and *syn* benzotris(norbornadiene), **60**. As shown in Scheme 21, the latter isomer underwent metathesis with a Grubbs catalyst to yield hexahydrosumanene, **61**, that was oxidised with DDQ to furnish the final product [37].

**Scheme 22:**
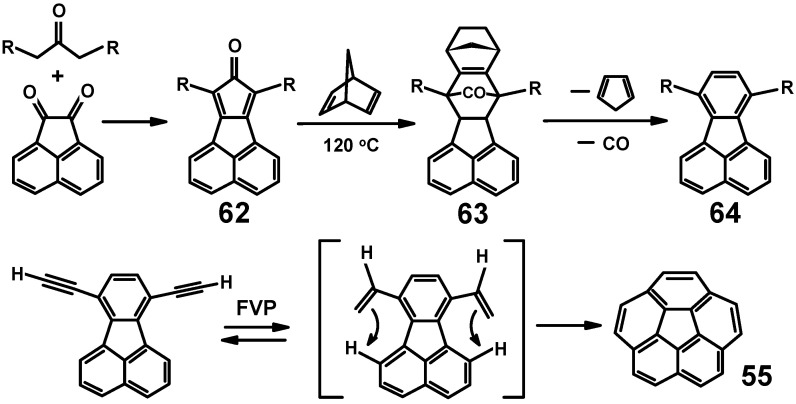
Scott’s original route to corannulene.

Corannulene was originally prepared in 1% yield in a heroic 17-step synthesis [38], and only became more readily available when Scott reported a route involving three benzannulations starting from acenaphthenequinone. Knoevenagel condensation to form the cyclopentadienone **62** was followed by cycloaddition with norbornadiene to yield **63** that suffered a retro-Diels–Alder elimination of cyclopentadiene and loss of CO, thereby generating the fluoranthene derivative, **64**, bearing two ethynyl substituents (Scheme 22). Finally, FVP brought about isomerisation (presumably in a stepwise manner) to the vinylidene intermediate enroute to corannulene [39].

**Scheme 23:**
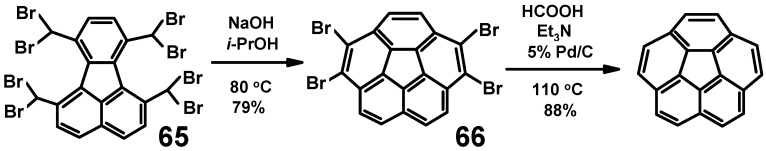
Key steps in Siegel’s kilogram-scale synthesis of corannulene.

This approach has since been modified and improved by Siegel, and some of the final steps are shown in Scheme 23. The conversion of the tetrakis-dibromomethylated precursor, **65**, via tetrabromocorannulene, **66**, to corannulene itself avoids the FVP step and, very impressively, has been carried out on the kilogram scale [40,41].

**Scheme 24:**
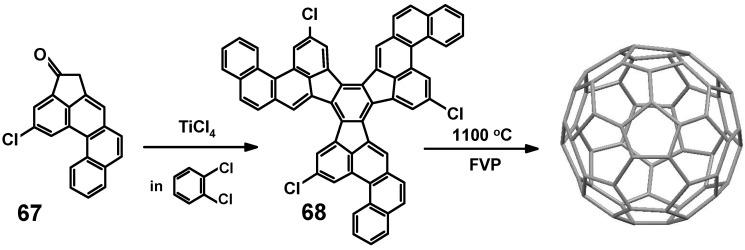
A rational synthesis of C_60_.

Knowing that acid-catalysed trimerization of indanone yields truxene, Scott and De Meijere undertook the titanium-mediated cyclisation of the pentacyclic ketone, **67**, which led to the *C*_3h_-symmetric molecule, **68** (Scheme 24). Under FVP conditions, this system with its 16 rings and 60 carbons suffered multiple dehydrohalogenations and dehydrogenations, thereby “stitching together” its three arms to form C_60_ (albeit only in very low yield) that was characterised by mass spectrometry [42].

### 5.2. Synthetic Routes to Benzazulenes

#### 5.2.1. Starting from Azulenes

**Scheme 25:**
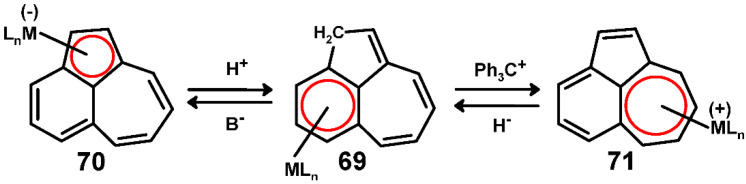
Possible haptotropic shifts over the benz[*cd*]azulene skeleton.

The benz[*cd*]azulene system with its fused five-, six- and seven-membered rings posed a tempting target with the possibility of haptotropic migrations among the rings dependent on the molecular charge. One could envisage a neutral η^6^ species, **69**, where deprotonation should yield the anionic η^5^ product, **70**, whereas removal of hydride might form a cationic η^7^ isomer, **71** (Scheme 25).

**Scheme 26:**
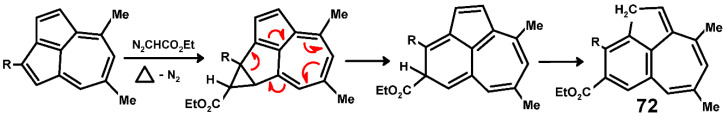
Hafner’s route to a 2*H*-benz[*cd*]azulene, **72**.

Early syntheses of a number of benzazulene isomers led to air-sensitive and thermally unstable materials in only modest yields. Typically, as shown in Scheme 26, Hafner and Rieper reported that treatment of cyclopent[*cd*]azulene with ethyl diazoacetate, i.e., a carbene addition process, led ultimately to ring expansion of a five-membered ring and, upon tautomerisation of the initially formed product, furnished ethyl 7,9-dimethyl-2*H*-benz[*cd*]azulene-4-carboxylate, **72** [43].

**Scheme 27:**
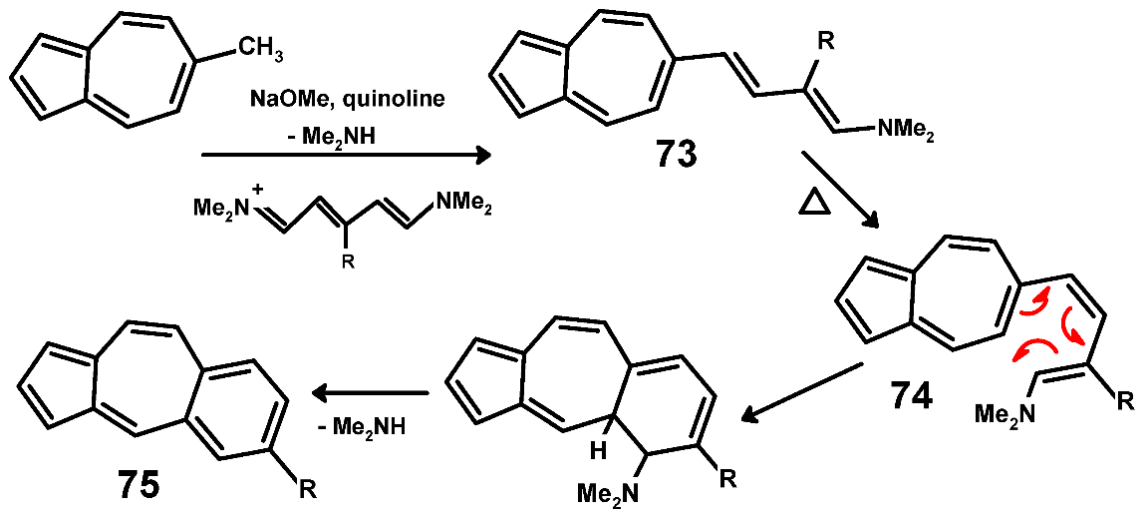
Synthetic route to benz[*f*]azulene **75**.

**Scheme 28:**
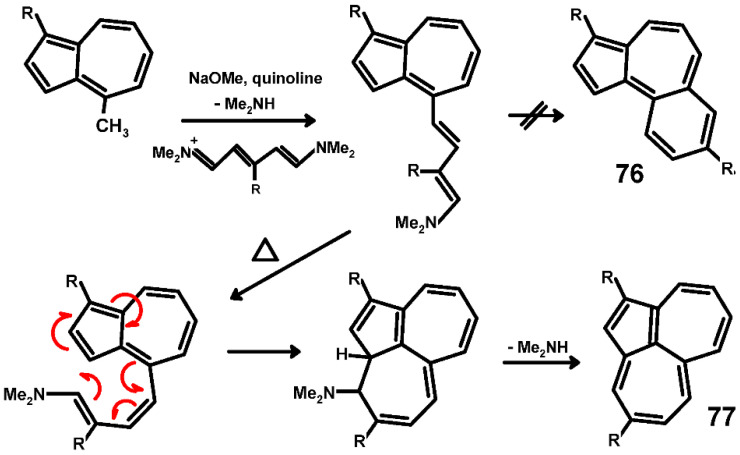
Attempted synthesis of the benz[*e*]azulene, **76**, led instead to the cyclopenta[*ef*]heptalene, **77**.

**Scheme 29:**
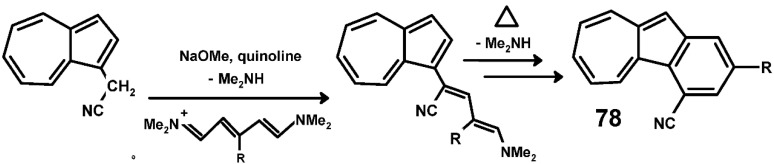
Synthetic route to a benz[*a*]azulene, **78**.

As shown in Scheme 27, Scheme 28 and Scheme 29, some years later, Jutz and Schweiger [44] found that, under basic conditions, 6-methylazulene and trimethinium perchlorate condense to generate the azulene-dienamine, **73**, that isomerised upon heating to form **74**, now poised for an electrocyclic ring closure and loss of dimethylamine to form ultimately the benz[*f*]azulene, **75**.

However, under the same conditions, the apparently closely analogous 4-methylazulene failed to deliver the anticipated benz[*e*]azulene, **76**, but instead yielded the isomeric cyclopenta[*ef*]heptalene, **77**. Nevertheless, continuing this approach, it was established that the analogous reaction with 1-azuleneacetonitrile led to the benz[*a*]azulene skeleton, **78** [44].

**Scheme 30:**
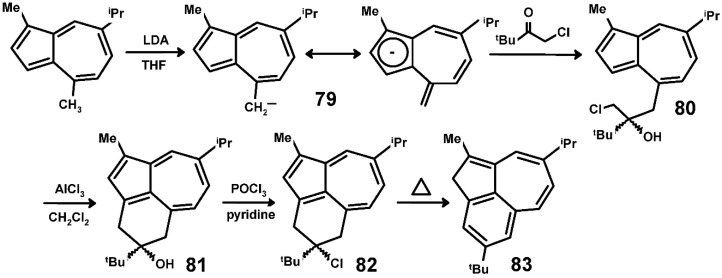
Synthesis of 2*H*-benz[*cd*]azulene, **83**, starting from guaiazulene.

Our own route to the 2*H*-benz[*cd*]azulene framework started from the readily available precursor guaiazulene, once again taking advantage of the facile deprotonation of the 4-methyl group due to delocalisation of the negative charge into the cyclopentadienide ring (Scheme 30). Nucleophilic addition of the resonance-stabilised anion, **79**, to 1-chloropinacolone led to the alcohol **80**, as a blue oil. AlCl_3_-promoted Friedel–Crafts cyclisation to form the six-membered ring in **81** was followed by treatment with phosphorus oxychloride, thus generating **82**, which upon warming delivered the desired 2*H*-benz[*cd*]azulene, **83**, as an orange solid, in 60% yield [45].

**Figure 11:**
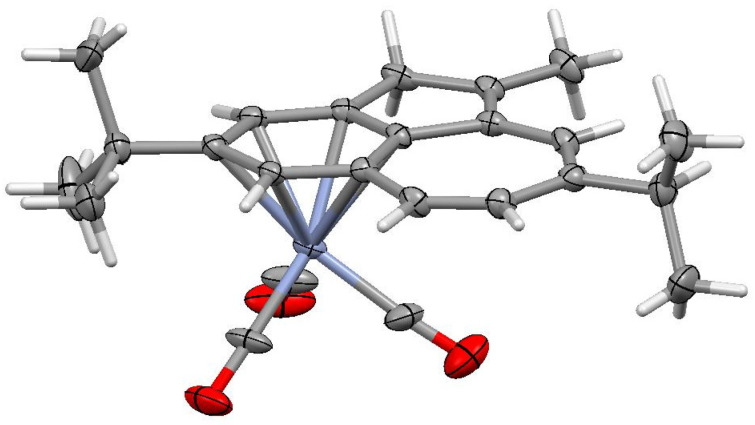
Molecular structure of the 2*H*-benz[*cd*]azulene-chromium tricarbonyl complex, **85**.

**Scheme 31:**
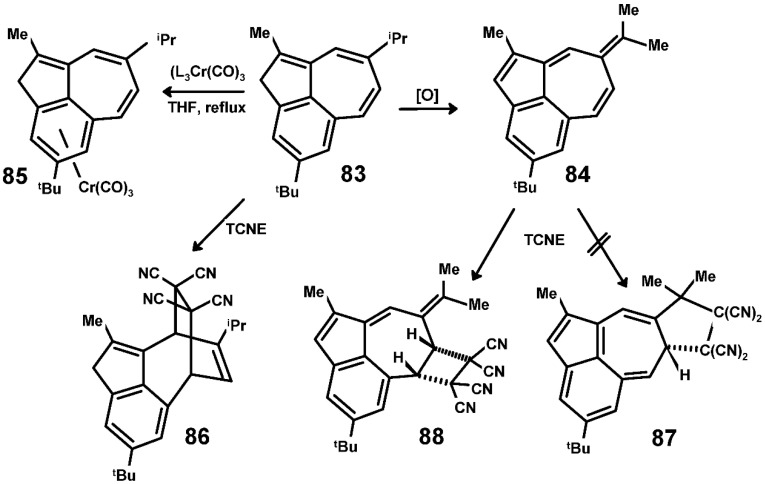
Reactions of benz[*cd*]azulenes **83** and **84** with tetracyanoethylene.

Chromatographic separation also yielded the yellow side-product **84**, (~10%) arising from partial aerial oxidation (Scheme 31). Reaction of **83** with (CH_3_CN)_3_Cr(CO)_3_ furnished the orange-red η^6^-Cr(CO)_3_ complex **85**, in which the planar character of the benz[*cd*]azulene framework was confirmed by X-ray crystallography (Figure 11).

It was also interesting to compare the cycloaddition chemistry of **83** and its oxidised counterpart, **84**, upon treatment with tetracyanoethylene. As depicted in Scheme 31, in the former case, the product was **86**, the anticipated [4 + 2] Diels–Alder adduct across the seven-membered ring. In the latter molecule, one might have considered the possibility of a [12 + 2] cycloadduct, **87**, requiring involvement of the external double bond; however, the reaction of TCNE with **84** led instead to [2 + 2] cycloaddition across the C(6)-C(7) bond, as in **88** [45].

**Scheme 32:**
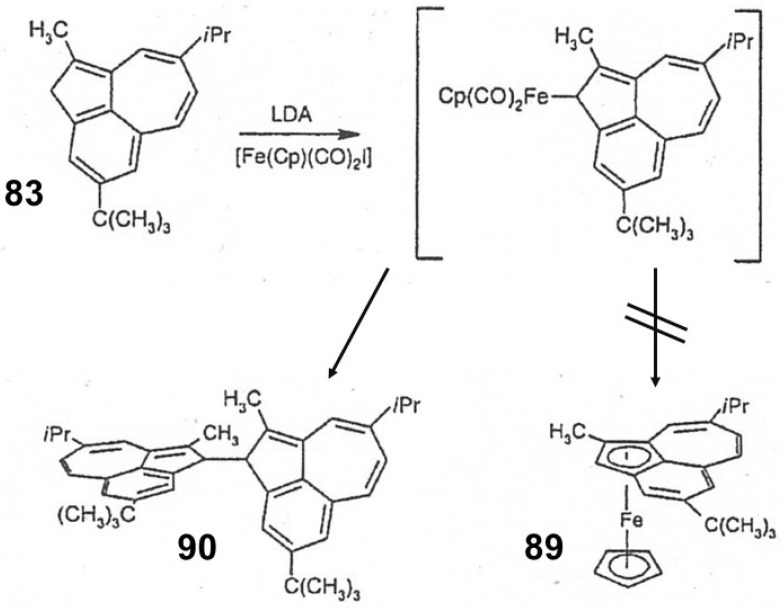
Attempted synthesis of a ferroceno-benzindene, **89**, led instead to a dimer **90**.

Finally, as shown in Scheme 32, an attempt to form the benz[*cd*]azulenyl-ferrocene, **89**, was unsuccessful. Instead, the product isolated was the dimer, **90**, that presumably arose upon thermolytic cleavage of the iron–carbon bond, leading to radical coupling—a situation paralleling the behaviour of 4*H*-cyclopenta[*def*]phenanthrene discussed in Section 3.2.1.

#### 5.2.2. Starting from Dibenzosuberenone

**Scheme 33:**
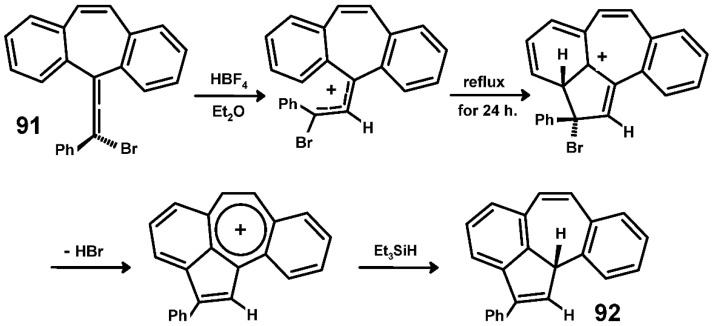
Preparation of 2-phenyl-11b*H*-dibenz[*cd*,*h*]azulene, **92**, from the bromoallene **91**.

In continuation of our studies on fluorenylidene-allenes [46], we chose to attempt the preparation of their seven-membered ring counterparts derived from dibenzosuberenone. The reaction of dibenzosuberenone with phenylethynyllithium to form the alkynol, followed by treatment with HBr, brought about rearrangement to the corresponding bromoallene, **91**, whose ready availability opened up a possible convenient route to the dibenz[*cd*,*h*]azulene system, as in Scheme 33. Indeed, protonation of **91** with HBF_4_ in diethyl ether occurs at the central carbon of the allene and, after heating at reflux for 24 h, cyclisation, elimination of HBr, and finally treatment with triethylsilane delivered 2-phenyl-11b*H*-dibenz[*cd*,*h*]azulene, **92**, as a white solid [47].

**Figure 12:**
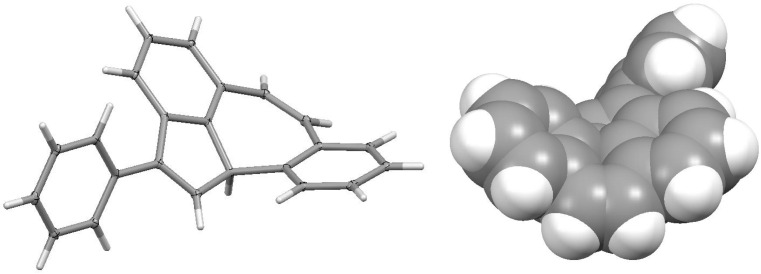
Molecular structure of 2-phenyl-11b*H*-dibenz[*cd*,*h*]azulene, **92**.

In contrast to the planar skeleton of 8-isopropyl-1-methyl-4-tert-butyl-2*H*-benz[*cd*]azulene, **83**, whereby the only sp^3^-type carbon is at C(2) in the five-membered ring, the presence of the hydrogen in a pseudo-axial position at C(11b) in **92** renders the molecule non-planar. The seven-membered ring adopts a boat conformation, whereas the indene unit is almost planar, and the 2-phenyl substituent is rotated through 41° relative to the five-membered ring. The space-filling representation shown as Figure 12 emphasises the bowl-shaped character of the molecule [47].

## 6. Serendipitous Formation of Tetracenes from 9-Phenylethynyl-9*H*-fluorene

**Scheme 34:**
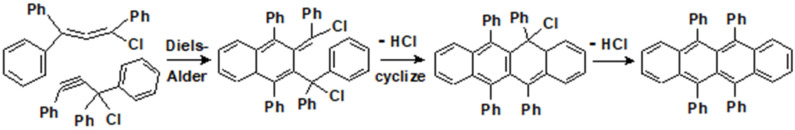
Original (1963) mechanistic proposal for the formation of rubrene.

Tetracenes are commercially significant on account of their photophysical properties, in particular their use as organic light-emitting diodes (OLEDs) in electroluminescent devices and in field-effect transistors (OEFTs) [48,49]. The classic example is that of 5,6,11,12-tetraphenyltetracene (rubrene), which is prepared by treatment of the acetylenic carbinol with HCl, and was originally postulated to rearrange to chloro-triphenylallene and then dimerise in a Diels–Alder process with subsequent elimination of HCl, as in Scheme 34 [50]. However, this proposed mechanism was superseded when it was revealed that allenes can undergo a variety of [2 + 2] cycloaddition processes to form bis(alkylidene)cyclobutanes via diradical biallyl intermediates [51].

**Scheme 35:**
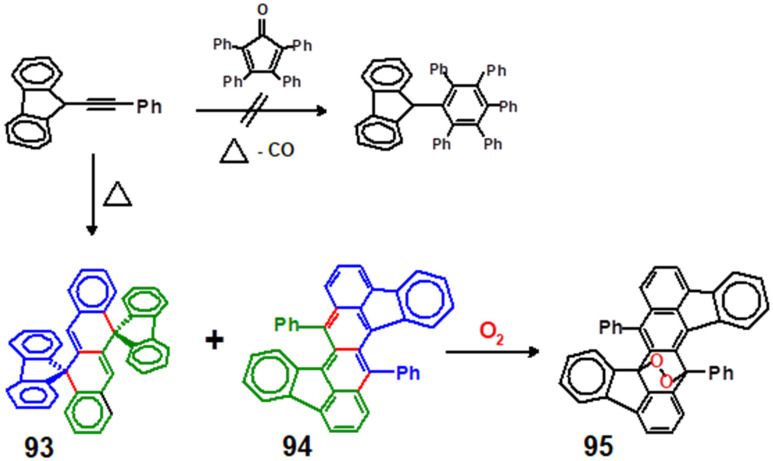
Unexpected formation of the dispirofluorenyltetracene, **93**, diindenotetracene, **94**, and peroxide, **95**.

**Figure 13:**
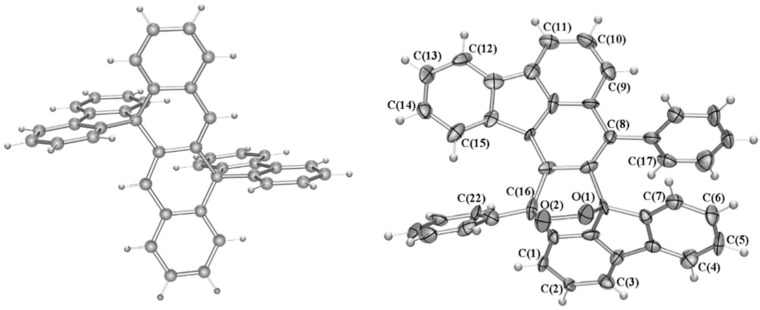
Molecular structures of dispirotetracene, **93**, and the tetracene peroxide, **95**.

Our involvement in this project was entirely serendipitous when the attempted Diels–Alder cycloaddition of 9-phenylethynyl-9*H*-fluorene to tetracyclone, with subsequent loss of carbon monoxide to form 9*H*-fluorenylpentaphenylbenzene, gave instead the pale yellow dispirofluorenyltetracene, **93**, the deep blue diphenyl-di-indenotetracene, **94**, and the peroxide **95** resulting from the aerial oxidation of **94**; the tetracyclone was recovered unchanged [52]. As illustrated in Scheme 35, these products are clearly derived from two molecules of the original alkyne, and were all unambiguously characterised by X-ray crystallography, as shown in Figure 13.

**Scheme 36:**
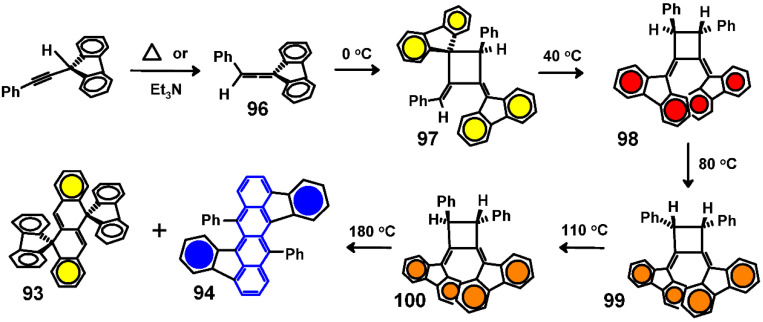
Isomerisation of phenethynylfluorene to allene, **96**, dimerisation to form a series of diphenyl-bis(fluorenyliden)cyclobutanes, and ultimately the tetracenes **93** and **94**.

Further study revealed that the alkyne had isomerised to the allene, **96**, which dimerised and progressed via a series of highly coloured (yellow, red, orange) bis(fluorenyliden)cyclobutanes and finally formed the tetracenes. When this reaction was carried out at 0 °C, the only product was the yellow head-to-tail dimer identified as the bis(alkylidene)cyclobutene **97**. However, in refluxing tetrahydrofuran, this molecule underwent rearrangement to the red tail-to-tail isomer trans-3,4-diphenyl-1,2-bis(fluorenylidene)cyclobutene, **98**, in which the adjacent fluorenylidenes with their large (8.7 Å) wingspans overlapped such that their helical sense paralleled that of the phenyls. Furthermore, at 80 °C, **98** isomerised cleanly into orange cis-3,4-diphenyl-1,2-bis(fluorenylidene)cyclobutene, **99**, and then at 110 °C into orange **100** in which the helicity of the overlapping fluorenylidenes opposed that of the trans-phenyls. Finally, thermolysis of the original head-tail isomer, **97**, at 180 °C delivered the previously observed tetracenes, **93** and **94**. Each successive product was characterised spectroscopically and by X-ray crystallography, and the entire sequence is depicted in Scheme 36 [53].

**Scheme 37:**
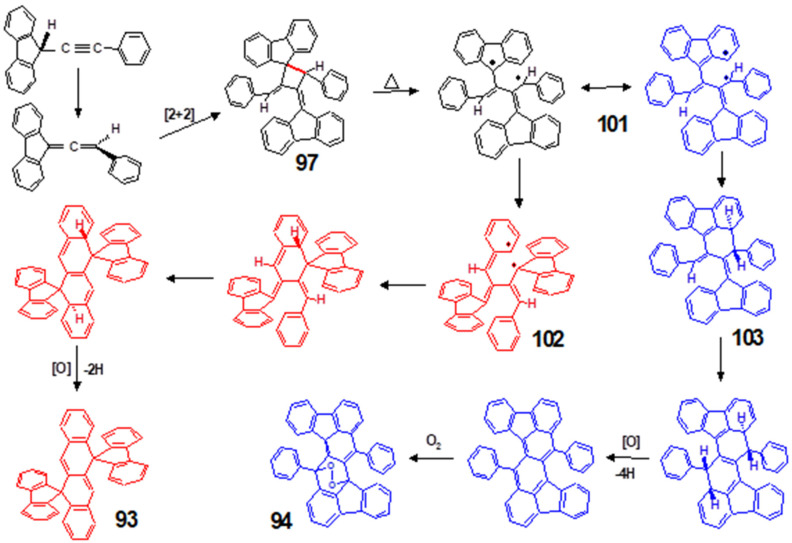
Proposed mechanism of the formation of tetracenes **93** and **94**.

The suggested mechanism is presented in Scheme 37 and involves initial isomerisation of the original alkyne to the allene **96**, which dimerises and suffers homolytic cleavage of the long (1.605 Å) bond in the four-membered ring of **97** to form the diradical **101**. Recombination can follow either the red or blue pathway to form six-membered rings, **102** or **103**, respectively; subsequent electrocyclization and finally oxidative dehydrogenation furnishes the tetracenes **93** and **94** [52].

**Scheme 38:**
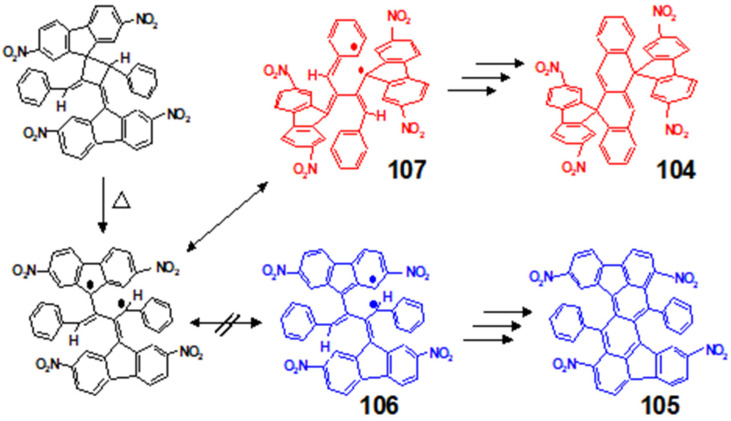
Preferential formation of dinitro-dispirotetracene **104**.

It is noteworthy that when 2,7-dinitrofluorenone is used instead of fluorenone to prepare the original alkyne, the major product found after thermolysis of 9-(phenylethynyl)-9*H*-2,7-dinitrofluorene at 180 °C is the dinitro-dispirotetracene, **104**, with only traces of the dinitro-diindenotetracene, **105** [54]. The preferential formation of **104** over **105** is presumably a reflection of the presence of the *meta*-nitro group, which is less than ideally situated to stabilise radical character localised on the *ortho*-carbon of the fluorenyl moiety in **106**. Hence, the more favoured process is ring expansion onto an *ortho*-carbon of the adjacent phenyl group in **107**, and subsequently leads, via the red pathway, to the corresponding dispirotetracene, as in Scheme 38.

## 7. Concluding Remarks

In this review, we have attempted to show the effects of benzannulation on a number of different molecular frameworks. Some of these examples involve well-established reactions, whereas others are of much more recent vintage, but their features in common are emphasised to give a more general overall picture. When indenes are generated from substituted cyclopentadienes, not only has the chiral character thus engendered been exploited for the elucidation of symmetry-allowed molecular rearrangements, but also their transition metal complexes have been utilised as catalysts for stereospecific polymerisations. The presence of the additional ring also features in cases whereby rates of reaction (substitutions, sigmatropic or haptotropic rearrangements) are substantially enhanced because of the temporary development of aromatic character in the transition state. Examples selected include benzannulations of indenes, fluorenes, and pentalenes. While benzynes generally undergo facile cycloadditions to linear acenes, their reactions with 3-(9-indenyl)anthracene and 2-phenylindene gave rise to novel adducts whose identity was only established unequivocally by X-ray crystallography.

Enroute to C_60_, multiple benzannulation has been elegantly accomplished whereby important fullerene fragments, such as sumanene or corannulene, have been prepared on a preparative useful scale, thereby allowing their chemistry to be thoroughly explored. Likewise, benzannulation of azulenes to furnish a range of isomeric benzazulenes has now progressed from the difficult early routes that led to thermally unstable products in low yields, to more readily available systems whose structures have been characterised spectroscopically and by X-ray crystallography. Finally, the mechanism of the stepwise conversion of fluorenyl-allenes to dispiro- and diindenyl-tetracenes has also been elucidated, and the numerous intermediates have been isolated and fully characterised by X-ray crystallography.
